# Systematic development of a training program for healthcare professionals to improve communication about breast cancer genetic counseling with low health literate patients

**DOI:** 10.1007/s10689-020-00176-3

**Published:** 2020-04-22

**Authors:** Jeanine A. M. van der Giessen, Margreet G. E. M. Ausems, Maria E. T. C. van den Muijsenbergh, Sandra van Dulmen, Mirjam P. Fransen

**Affiliations:** 1 Division Laboratories, Pharmacy and Biomedical Genetics, Department of Genetics, University Medical Center Utrecht, P.O. Box 85500, 3508 GA Utrecht, The Netherlands; 2 Pharos, Dutch Center of Expertise on Health Disparities, Utrecht, The Netherlands; 3 Department of Primary and Community Care, Research Institute for Health Sciences, Radboud University Medical Center, Nijmegen, The Netherlands; 4 Nivel (Netherlands Institute for Health Services Research), Utrecht, The Netherlands; 5 Faculty of Health and Social Sciences, University of South-Eastern Norway, Drammen, Norway; 6 Department of Public Health, Amsterdam Public Health Research Institute, Amsterdam UMC, University of Amsterdam, Amsterdam, The Netherlands

**Keywords:** Blended training program, Breast cancer genetic counseling, Communication skills, Disparities, Health literacy, Migrant status, Referral

## Abstract

There is a disproportionate underuse of genetic testing in breast cancer patients from lower education or migrant background. Within these groups, communication about referral to genetic counseling appears challenging due to limited health literacy and cultural barriers. Our aim was to develop and evaluate a training program for healthcare professionals (breast surgeons and specialized nurses), to increase effective communication. We systematically developed a blended training program based on patients’ and healthcare professionals’ needs and preferences. Prior to the training, we assessed awareness, knowledge and self-efficacy of healthcare professionals. Acceptability and usefulness of the training program were assessed directly after the training. Healthcare professionals (n = 65) from 17 hospitals showed moderate to high awareness and knowledge about the prevalence and impact of limited health literacy. They were aware of cultural factors that influence communication. However, they did not feel confident in recognizing limited health literacy and their self-efficacy to communicate effectively with these patients was low. The training program was rated as acceptable and useful. Healthcare professionals lack confidence to effectively communicate with patients with limited health literacy or migrant background. The training program offers opportunities to improve communication about referral to breast cancer genetic counseling.

**Electronic supplementary material:**

The online version of this article (10.1007/s10689-020-00176-3) contains supplementary material, which is available to authorized users.

## Introduction

Referral to genetic counseling for breast cancer patients at risk of carrying a mutation is crucial and should preferably be offered early after diagnosis to guide treatment decisions. Breast cancer patients with a BRCA 1/2 gene mutation can decide whether or not to opt for bilateral mastectomy as primary surgery and also the chemotherapeutic approach for these patients can be different [1–3]. In addition, an abnormal test result may have important implications for cancer prevention strategies for patients and their (healthy) family members, including future generations. Although genetic counseling is clinically relevant for all eligible high-risk patients with breast cancer, there’s still a disproportionate underuse of it in patients with a lower educational background and in migrant patients [4–7]. These patients seem to have poorer access to cancer-related genetic counseling [7, 8]. Patients need to understand the benefits, limitations and risks of genetic testing, value this information, communicate about it properly with healthcare professionals and family members and make an informed decision regarding the possible consequences of a genetic test result. This requires adequate health literacy, which is generally defined as a persons’ ability to access, understand, evaluate and use health-related information and is recognized as a critical factor affecting communication in cancer care [9]. Research shows that poor awareness of family history, inaccurate risk perception and a lack of awareness of genetic services contribute to patients’ misunderstanding of genetic services [10, 11]. Besides, patients with limited health literacy also show a lower preference for active participation in decision-making about genetic testing [12] and in taking initiative for referral to genetic counseling. For migrant breast cancer patients, language difficulties and limited health literacy, as well as cultural factors, are determinants for non-participation in genetic counseling [13].

However, physicians also contribute to these disparities in access to breast cancer genetic counseling in the way they communicate. Provider recommendation is a first step towards uptake of genetic counseling [14] but referral is not always adequately discussed with patients with limited health literacy [15–18]. Women attribute their low levels of awareness of genetic testing to a lack of physicians’ recommendation for referral, which they also noted as their primary reason for not receiving testing [17, 19]. Baars et al. showed that a major cause for the low participation rate in cancer genetic counseling lies within the referral process. Although referral guidelines are sufficiently available and known by physicians, they do not always act in concordance with these guidelines [20, 21]. Gaps in effective communication are widely recognized as a major contributor to health disparities [22] also in the genetic testing [17]. Employing effective communication techniques for healthcare professionals is an important intervention to reduce health disparities related to limited health literacy [23]. However, implementation of a training program for healthcare professionals is a complex process. Successful adoption is only possible if healthcare professionals themselves deem it useful [24]and when they are involved during the development of the program [25]. The aim of the present study was to develop a training program for healthcare professionals (breast surgeons and specialized nurses) to communicate effectively about referral to breast cancer genetic counseling with patients with limited health literacy or a migrant background. Specific objectives were: 1. to develop a training program based on the needs and preferences of healthcare professionals and patients, 2. to assess knowledge, awareness and self-efficacy in communication with patients with limited health literacy or a migrant background, and 3. to gain insight in the usefulness and acceptability of the training program from healthcare professionals’ perspective.

## Materials and methods

### Development of the training program

We systematically developed a blended training program (Erfo4all), consisting of an online module and a group training, based on healthcare professionals’ and patients’ needs and preferences. The intervention mapping (IM) approach [26] a protocol for developing theory- and evidence-based health education programs was used as a helpful guideline (Table 1 in Supplementary Materials).

Based on (a) the needs and preferences of healthcare professionals and patients and insights from our previous studies and (b) a matrix of change performances and objectives, we (c) made deliberate choices regarding the design and content of the training program and (d) pilot-implemented the program in clinical practice. Each step in the development process is described in detail below.

#### Assess needs and preferences of healthcare professionals and patients

We conducted a group interview with breast surgeons and specialized nurses, who are the main referrers to genetic counseling for patients with breast cancer [8, 27] to assess their preferences regarding content and design of a training program and to gain insight into conditions to participate. They were recruited from different breast cancer teams from different hospitals in Western Netherlands. For the content of the training program from patients’ perspective we elaborated on findings from our previous study on participation determinants and perspectives of (migrant) patients and healthcare professionals in breast cancer genetic counseling [13, 20]. In the present study we conducted in-depth interviews with three patients to deepen the relevance of our findings. Patients were asked to share their experience with breast cancer genetic counseling and state barriers, needs and preferences for communication with their surgeon or specialized nurse. They were recruited in collaboration with Mammarosa, an organization that provides information about breast cancer for migrant patients and patients with a low level of literacy. The patients were able to speak Dutch and had personal experience with breast cancer care and cancer genetic counseling. The interviews with healthcare professionals and patients were audio-recorded and transcribed verbatim to increase validity. Analyses were done by two authors, working independently. Seven healthcare professionals (three breast surgeons, one medical oncologist and three specialized nurses) participated in the group interview. The group interviews indicated that healthcare professionals experience difficulties in recognizing patients with limited health literacy in daily practice and a tendency to overestimate the health literacy skills of their patients. They also indicated a need for more information and for tools to communicate effectively about referral to breast cancer genetic counseling with patients with limited health literacy or a migrant background, Healthcare professionals had a clear preference for a blended learning intervention, consisting of an online module followed by a multidisciplinary group training of limited duration to enhance their skills in tailoring communication about genetic testing to patients with limited health literacy. Patients noted communication with the physician or specialized nurse as the most important factor influencing referral. They stated that the use of plain language, non-medical jargon and tailored information are very important in communication about breast cancer genetic counseling. Patients mentioned various difficulties with taking initiative for referral to breast cancer genetic counseling. They experienced insufficient knowledge and skills to discuss referral possibility with their physician when they were diagnosed with breast cancer. Asking questions was considered to be difficult for most migrant patients. According to patients, taking into account social and cultural beliefs about cancer and also the use of a professional interpreter contribute to more effective communication about referral to breast cancer genetic counseling.

#### Matrix of change performances and objectives

Based on healthcare professionals’ training preferences and input from patients’ perspective on the content of the training we specified performance and change objectives in a matrix of change (Table 2 in Supplementary Materials).

We then selected various practical strategies from literature to improve communication with limited health literate patients, such as information transfer to enhance knowledge and awareness about the problem of limited health literacy [28] and the Teach-back method to identify patients with limited health literacy [23, 29]. Role-play [30] was selected as a strategy to acquire required communication skills and to practice the use of plain language and the Teach-back method. To further enhance health professionals’ ability to communicate in an effective manner with patients with a migrant background, we selected strategies to enhance cultural competences [31].

#### Design and content of the training program

In the next step, the practical strategies were incorporated into a blended training program (Erfo4all), consisting of two successive parts: an online module (18 min) and a group training (2 h). The online module focused on knowledge acquisition, while in the group training practicing skills were most important. An online module offers opportunities to increase knowledge, but it is likely not sufficient for behavior change. Integrating an online training with traditional face-to-face training gives the opportunity to increase knowledge as well as practical skills. The background information in the training program was based on the reports on health literacy from the national institute for health services research in the Netherlands [32, 33]. We used video-recordings from the Dutch Reading and Writing foundation to include patients’ perspective in the background information. In these video-recordings low literate people shared their experience with being low literate, talked about their shame and explained how they tried to hide their problem in real life. Information about the prevalence of low literacy and limited health literacy in the Netherlands, the relevance of health literacy in understanding and appraising information from healthcare professionals as well as the way health literacy relates to socio-economic and demographic characteristics were incorporated in the online module. Also specific attention was given to communication with patients with a migrant background, including the impact of a language barrier and cultural factors on communication with healthcare professionals. The training methods were developed in collaboration with Pharos (Dutch Centre of Expertise on Health Disparities). We made use of their group training on effective communication with patients with limited health literacy or a migrant background and adapted it to the context of clinical genetics and breast cancer genetic counseling. Roleplay and the teach-back method already were key elements in their training and were further refined to reach our performance objectives. In cooperation with clinical geneticists, we added real-life cases, with a focus on migrant and non-migrant patients, in relation to cancer genetic counseling.

#### Pilot-implementation of the training program

We pilot-implemented the Erfo4all training program in 17 hospitals in three regions in the Netherlands. Healthcare professionals from these hospitals refer breast cancer patients for genetic counseling to clinical geneticists of one of three academic centers. Together with clinical geneticists from these three contributing academic centers, we developed a detailed plan on recruitment for breast surgeons and specialized nurses in referring hospitals, including instructions for contact persons to motivate colleagues to participate in the training. The Center for Research and Development of Education from the University Medical Center Utrecht created private accounts for the participants of the training program, which gave them access to a questionnaire and the online module. Accreditation by the Dutch Association for Surgery and the Dutch Professional Nurse Practitioner Organization was an incentive for participation.

### Assessment of knowledge, awareness and self-efficacy

Before the training, we assessed healthcare professionals’ knowledge, awareness and self-efficacy regarding communication with patients with limited health literacy or a migrant background using an online questionnaire. Knowledge was assessed with five multiple choice questions focusing on:

- prevalence of low literate adults in the Netherlands. Answers ranging from (A) to (D).
- limited (health) literacy in relation to people with a migrant background. Answers ranging from (A) to (C).
- prevalence of adults with limited health literacy in the Netherlands. Answers ranging from (A) to (D).
- level of education in relation to the level of health literacy. Answers ranging from (A) to (C).
- use of a professional interpreter (self- reported). Answers (yes) or (no).

Each item was rated as correct (1) or wrong (0) and a total knowledge score was computed as the number of correct answers.

Awareness was assessed by three items on:

- prevalence and impact of health literacy in the Netherlands
- impact of health literacy on medical communication
- importance to take into account cultural factors in communication with patients with a migrant background.

Each item was scored on a 5 point Likert scale ranging from (1) low, to (5) very high.

Self-efficacy was assessed by five statements on having confidence in:

- recognizing limited health literacy in patients
- communicating effectively about breast cancer genetic counseling with patients with limited health literacy
- understanding which customs and habits from patients with a migrant background might influence communication
- coping with cultural factors in communication with patients with a migrant background
- coping with a language barrier

Each item was scored on a 5 point Likert scale ranging from (1) totally disagree to (5) totally agree. Descriptive statistics were used to describe baseline characteristics and outcome variables using SPSS version 24.0 (SPSS, Chicago, IL). Data was used as baseline measurement for our study on effectiveness of the training program.

### Test training program on acceptability and usability

After completing the questionnaire healthcare professionals got access to the online module and within two weeks they were invited for the group training on location. Each healthcare professional completed a paper-and-pencil evaluation survey after completion of the training program. The evaluation survey contained five questions assessing acceptance of the program, measured on a 5-point Likert scale ranging from (1) very good to (5) not good at all. The following items were assessed:

- design of the online module.
- duration of the online module.
- blended learning method.
- duration of the group training.
- time schedule of the group training.

In addition, participants were asked to rate the usefulness of the training program measured on a five point Likert scale, ranging from (1) very useful to (5) not useful at all. The following items were assessed:

- online module.
- group training on location.
- training elements.
- recognizing low literacy/limited health literacy (teach-back method)
- general advice to communicate in plain language
- obtaining family history
- cultural sensitive communication
- specific advice to communicate in plain language about (referral to) breast cancer genetic counseling
- practicing real life cases (role-play)

Finally the quality of the module and the group training, as well as competence of the trainer and the training actress were rated on a scale from 1 (low)–10 (high).

## Results

### Response and characteristics of participants of the training

A total of 73 healthcare professionals were included in the training program. The online questionnaire was completed by 65 healthcare professionals from 17 hospitals. Table 1 shows an overview of background characteristics.

**Table 1:** Background characteristics of healthcare professionals (n = 65)

Variable	N	%	SD
**Sex**			
Male	12	18.5%	
Female	53	81.5%	
**Discipline**			
Breast surgeon	21	32.3%	
Specialized nurse	38	58.5%	
Medical oncologist	1	1.5%	
Physician assistant	2	3.1%	
Other	3	4.6%	
**Age**			45.7 (8.5)
**Work experience in breast cancer care (in years)**			10.9 (7.0)
**Clinical setting**			
University hospital	6	9.2%	
Community hospital	59	90.8%	

### Awareness, knowledge and self-efficacy healthcare professionals

Prior to the training, the majority of healthcare professionals showed a moderate to high score on awareness about the prevalence and impact of health literacy in the Netherlands, as well as on the impact of limited health literacy on medical communication (Table 2). They were highly aware of the importance to take into account cultural factors in the communication with patients with a migrant background and 46% reported to deploy a professional interpreter in communication with patients with a language barrier. Knowledge about prevalence of limited (health) literacy in the Netherlands was moderate (mean accurate knowledge score was 2.48, SD. 98) and most healthcare professionals knew which factors are related to limited health literacy. Self-efficacy in communication with patients with limited health literacy or a migrant background however, was low. Healthcare professionals reported to frequently encounter challenges in recognizing limited health literacy in patients, to communicate effectively about breast cancer genetic counseling and to cope with cultural factors in the communication with patients with a migrant background.

**Table 2:** Awareness, knowledge and self-efficacy prior to the training

	N	%
**Awareness**		
***Awareness of prevalence and impact of health literacy***		
Low	–	–
Barely	4	6.2%
Reasonably	49	75.4%
High	10	15.4%
Very high	2	3.1%
***Awareness of impact health literacy on communication***		
Low	–	–
Barely	3	4.6%
Reasonably	33	50.8%
High	27	41.5%
Very high	2	3.1%
***Awareness of the importance to assess cultural factors***		
Low	1	1.5%
Barely	1	1.5%
Reasonably	10	15.4%
High	42	64.6%
Very high	11	16.9%
**Knowledge**		
***Prevalence of illiteracy in the Netherlands***		
Correct answer	46	70.8%
Wrong answer	19	29.2%
***Limited (health) literacy and a migrant background***		
Correct answer	38	58.5%
Wrong answer	27	41.5%
***Prevalence adults with limited health literacy***		
Correct answer	20	30.8%
Wrong answer	35	55.4%
***Level of education related to level of health literacy***		
Correct answer	48	73.8%
Wrong answer	17	26.2%
***Use of professional interpreter***		
Correct answer	58	89.2%
Wrong answer	7	10.8%

Sum scores/total knowledge		Mean (SD)
1	2	3.1% 2.48 (.98)
2	14	21.5%
3	21	32.3%
4	23	35.4%
5	5	7.7%
**Self-efficacy**		
***Having confidence in understanding which customs and habits from patients with a migrant background might influence communication***		
Totally disagree	2	3.1%
Disagree	28	43.1%
Not agree/not disagree	27	41.5%
Agree	7	10.8%
Totally agree	1	1.5%
***Having confidence in recognizing limited health literacy***		
Totally disagree	–	–
Disagree	9	13.8%
Not agree/not disagree	30	46.2%
Agree	26	40.0%
Totally agree	–	–
***Having confidence in communicating effectively about breast cancer genetic testing with patients with limited health literacy***		
Totally disagree	–	–
Disagree	17	26.2%
Not agree/not disagree	36	55.4%
Agree	12	18.5%
Totally agree	–	–
***Having confidence in coping with cultural factors***		
Totally disagree	–	–
Disagree	16	24.6%
Not agree/not disagree	29	44.6%
Agree	20	30.8%
Totally agree	–	–
***Having confidence in coping with language barriers***		
Totally disagree	–	–
Disagree	9	13.8%
Not agree/not disagree	30	46.2%
Agree	26	40.0%
Totally agree	–	–

#### Acceptability and usefulness of the training program

**Fig. 1:**
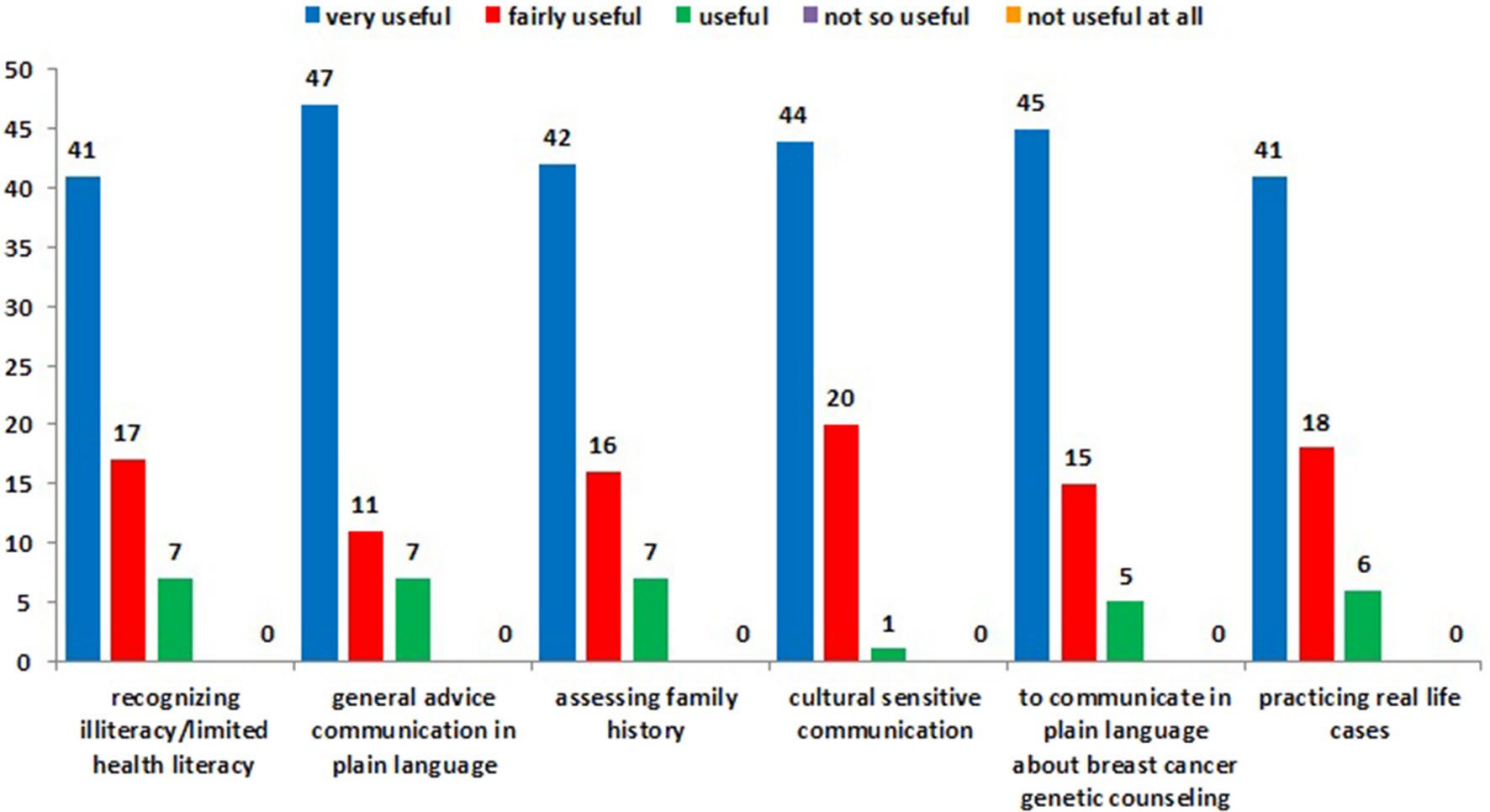
Perceived usefulness of training elements

The training program was evaluated positively by the healthcare professional. They reported a high degree of acceptance with the blended learning method; the combination of an online module and a group training on location was considered useful and time-efficient. They were satisfied with the duration of the training, both the module and the group training, as well as with the design of the module. Furthermore, the healthcare professionals appreciated the trainer and the training actress, the average score for the training actress was 9.3 and for the trainer 9.0 on a scale from 1 to 10. Figure 1 shows perceived usefulness of training elements. Training elements with a high score included recognizing low literacy/limited health literacy, general advice on how to communicate in plain language, assessing family history, cultural sensitive communication, communication about breast cancer genetic counseling in plain language and practicing with real-life cases. Most healthcare professionals would recommend the training to their colleagues. Overall, the participants’ evaluation suggests that the training program was well accepted.

## Discussion

In this paper we described the systematic development, pilot-implementation and acceptability of a blended training program for breast surgeons and specialized nurses to improve communication about referral to breast cancer genetic counseling. The content and format of the program was based on their training needs and preferences and tailored to patients’ perspective. Upon the training, healthcare professionals were aware of the problem of limited health literacy and reported to have knowledge about prevalence of low literacy, limited health literacy and the main factors associated with health literacy. However, they didn’t feel competent to recognize limited health literacy and to communicate effectively with these groups of patients. The training program was evaluated as acceptable on method, design and duration, and participants rated the digital module, group training and training elements as useful.

The needs and preferences that we obtained during the development of the training program, indicated that healthcare professionals experience difficulties in recognizing limited health literacy in patients and are in need of techniques to communicate effectively about referral to breast cancer genetic counseling with patients with limited health literacy or a migrant background. This need for training has been reported by others as well [34–36]. Coelho (2018), for example, found that 84% of the healthcare professionals would like more training on health literacy, including assessment tools and techniques to manage limited health literacy [34]. An unexpected finding of our study was that healthcare professionals’ knowledge and awareness regarding prevalence and impact of limited health literacy and cultural factors influencing communication was generally adequate. Other studies indicated lower perceived awareness and knowledge about health literacy [34, 36]. Although healthcare professionals seem generally aware of cultural differences, different studies indicate that awareness is not enough. Enhancing cultural competence, the ability to cope with cultural differences, is important to communicate effectively with patients with a migrant background [37, 38]. The outcomes of the questionnaires further indicated that healthcare professionals’ self-efficacy to communicate with patients with limited health literacy or a migrant background was low. Therefore, improvement of healthcare professionals’ self-efficacy to communicate with patients with limited health literacy or a migrant background is important, especially because self-efficacy is related to one’s competence and to future (communication) behavior. Knowledge alone is insufficient for actual behavior change. Therefore, using role play, focusing on plain language, using the teach-back method and cultural sensitive communication, are key elements in our training program. We choose for role play because this is an effective training strategy to practice and learn communication skills [39]. Other studies showed promising results regarding the use and effectiveness of the teach-back method in communication with patients with limited health literacy [40] and cultural sensitivity training for improved understanding of cultural factors and the ability to communicate with patients with a migrant background [31]. Participants in our study were very positive about the acceptability and usability of the training, this is important for adoption and successful implementation of the program. Implementation effectiveness is critical for transporting interventions to daily practice [41]. Because of the high acceptance of the program and focus on enhancing skills, the Erfo4all training program seems to offer opportunities to improve communication about breast cancer genetic counseling. The setting of breast cancer genetic counseling is not unique compared to genetic counseling for other types of cancer or even genetic disorders. In general, limited health literacy is associated with lower genomic related knowledge and it affects patients’ understanding of print and oral communications about genetic and genomic information, so adapting communication to patients with limited health literacy is important in different settings of genetic counseling. We think is feasible to adapt our program to these other settings. The next step in our research is to study the effectiveness of the Erfo4all training program on knowledge, awareness and self-efficacy regarding communication with patients with limited health literacy or a migrant background.

## Strength and limitations

A strength of this study was the systematic approach in the development of the training. The needs and preferences of healthcare professionals and patients were used to determine the format and content of the program and to enhance a successful implementation. However, there are also some potential limitations. First, we included healthcare professionals in the training program on a voluntary base, so selection bias cannot be ruled out. Healthcare professionals who are already more aware of the problem of limited health literacy and have a basic knowledge about the subject, may be more interested in participating in the training program. And second, we assessed awareness and self-efficacy by a self-reported instrument, so bias, like social desirability, may affect the results. Our study emphasizes the need and feasibility of a training program for healthcare professionals in the context of clinical genetics and can be used to improve communication about breast cancer genetic counseling with patients with limited health literacy or a migrant background. We are currently performing a study to find out whether this training program contributes to a higher referral rate and increased access to breast cancer genetic testing for these groups of patients. In this study we specifically developed a training program for healthcare professionals and not for patients. In future research it may be worthwhile to consider whether empowering patients (e.g., by asking questions, or by taking the initiative to discuss possible genetic causes of their breast cancer) can also contribute to effective communication about referral to breast cancer genetic counseling.

## Electronic supplementary material

Below is the link to the electronic supplementary material.

Supplementary file1 (DOCX 14 kb)

Supplementary file2 (DOCX 15 kb)
