# Supplementary material for: Systematic development of a training program for healthcare professionals to improve communication about breast cancer genetic counseling with low health literate patients
Source: Fam Cancer. 2020 Apr 22;19(4):281–90. doi: 10.1007/s10689-020-00176-3 (PMC7497313; doi:10.1007/s10689-020-00176-3)
Supplement: Supplementary file 1 — Supplementary file1 (DOCX 14 kb) [file 10689_2020_176_MOESM1_ESM.docx]

*Table 1. Steps in systematic development of the training program; intervention mapping approach*

| **Step** | **Task** |
| --- | --- |
| 1. needs assessment | specify needs of healthcare professionals  (breast surgeons and specialized nurses)  specify needs of patients |
| 2. definition of performance and change objectives | establish a matrix of change |
| 3. selection of intervention, methods and strategy | determine focus of the intervention |
| 4. design and production of the intervention | design of the training program |
| 5. implementation plan | develop an implementation plan to examine the effect of the training program |
| 6. evaluation plan | describe methods for evaluation of the intervention |
