# Supplementary material for: Systematic development of a training program for healthcare professionals to improve communication about breast cancer genetic counseling with low health literate patients
Source: Fam Cancer. 2020 Apr 22;19(4):281–90. doi: 10.1007/s10689-020-00176-3 (PMC7497313; doi:10.1007/s10689-020-00176-3)
Supplement: Supplementary file 2 — Supplementary file2 (DOCX 15 kb) [file 10689_2020_176_MOESM2_ESM.docx]

Table 2. Matrix of change, performance objectives and change objectives in relation to communication about (breast) cancer genetic counseling

| **Performance**  **objectives** | **Determinants** | | | |
| --- | --- | --- | --- | --- |
|  | *awareness* | *knowledge* | *attitude* | *skills* |
|  | *change objectives* | | | |
| **Health professionals recognize signs of low literacy or limited health literacy in patients** | Health professionals are aware of the fact that many people in the Netherlands have difficulties with reading, understanding and processing (medical) information. | Health professionals know which (group of) patients are most at risk in being low (health) literate  Health professionals know which communication techniques are most appropriate in order to detect low literacy or limited health literacy. | Health professionals consider it important to recognize low literacy or limited health literacy in patients. | Health professionals can apply specific communication techniques to detect low literacy or limited health literacy in patients. |
| **Health professionals communicate effectively about breast cancer genetic counseling with patients with low literacy or limited health literacy** | Health professionals are aware of the differences in referral to cancer genetic counseling between women with a low educational background or migrant patients and women with a high educational background.  Health professionals are aware of their (shortcomings in) communication skills. | Health professionals know how to explain information about breast cancer genetic counseling in plain Dutch to patients with low literacy, limited health literacy or a migrant background. | Health professionals consider it important to tailor their communication and explain information about breast cancer genetic counseling in plain Dutch. | Health professionals adapt their communication to the needs and abilities of the patient and his or her family. |
| **Health professionals adequately cope with a language barrier** | Health professionals are aware of the impact of a language barrier on medical consultation. | Health professionals know why a professional interpreter is preferable to a family interpreter.  Health professionals know how to access the services of a professional interpreter. | Health professionals have a positive attitude toward the use of a professional interpreter instead of family interpreter. | Health professionals make adequate use of the services of a professional interpreter. |
| **Health professionals act in a culturally sensitive manner** | Health professionals are aware of their knowledge and beliefs about people with a different cultural background. | Health professionals know how to take into account different cultural, psychological and religious aspects in their communication with patients with a migrant background and their family members. | Health professionals consider it important to take into account cultural aspects. | In their communication with patients with a migrant background, health professionals take into account cultural aspects, such as a taboo on talking about cancer. |
